# Toxic elements and bio-metals in *Cantharellus* mushrooms from Poland and China

**DOI:** 10.1007/s11356-017-8554-z

**Published:** 2017-03-18

**Authors:** Jerzy Falandysz, Maria Chudzińska, Danuta Barałkiewicz, Małgorzata Drewnowska, Anetta Hanć

**Affiliations:** 1 Laboratory of Environmental Chemistry and Ecotoxicology, Gdańsk University, 63 Wita Stwosza Str, 80-308 Gdańsk, PL Poland; 2 Department of Trace Element Analysis by Spectroscopy Method, Adam Mickiewicz University, Umultowska 89b, 61-614 Poznań, Poland; 3 Rozany Strumien Base Station of Integrated Monitoring of Natural Environment, Faculty of Geographical and Geological Sciences, ul. Bogumiła Krygowskiego 10, 61-680 Poznań, Poland

**Keywords:** Mushrooms, Minerals, Organic food, Trace elements, China, Poland

## Abstract

Data on multi-trace element composition and content relationships have been obtained for *Cantharellus cibarius*, *C. tubaeformis*, and *C. minor* mushrooms from Poland and China by inductive coupled plasma–dynamic reaction cell–mass spectroscopy. There is no previous data published on As, Li, V, Tl, and U in chanterelles from Poland and on Ba, Co, Cr, Ni, Rb, and Sr in chanterelles from China. The results implied a role of the soil background geochemistry at the collection site with the occurrence of Ag, As, Ba, Cr, Cs, Li, Mn, Pb, Rb, Sr, U, and V in the fruiting bodies. Both geogenic Cd and anthropogenic Cd can contribute in load of this element in chanterelles from the Świetokrzyskie Mts. region in Poland, while geogenic source can be highly dominant in the background areas of Yunnan. An essentiality of Cu and Zn and effort by mushroom to maintain their physiological regulation could be reflected by data for *Cantharellus* mushrooms from both regions of the world, but its geogenic source (and possibly anthropogenic) can matter also in the region of the Świetokrzyskie Mountains in Poland. The elements Co, Ni, and Tl were at the same order of magnitude in contents in *C. cibarius* in Poland and Yunnan, China. *C. tubaeformis* differed from *C. cibarius* by a lower content of correlated Co, Ni, and Zn. Soil which is polymetallic and highly weathered in Yunnan can be suggested as a natural geogenic source of greater concentrations of As, Ba, Cr, Li, Pb, Sr, U, and V in the chanterelles there while lower of Mn and Rb, when related to chanterelles in Poland. A difference in Cs content between the sites can be attributed as an effect of the ^137^Cs release from the Chernobyl accident, in which Poland was much more affected than Yunnan, where deposition was negligible.

## Introduction

Mushrooms from the genus *Cantharellus* are well recognizable and are a popular food item in many regions of the world, e.g., *C. cibarius* Fr. The common names for *C. cibarius* are chanterelle, common chanterelle, golden chanterelle, or girolle. Advances in analytical methods and instrumentation enabled in recent years better insight into the mineral constituents of mushrooms (Dimitrijevic et al. 2016; Gąsecka et al. 2017; Mleczek et al. 2017; Stefanović et al. 2016a, b).

Wild-growing mushrooms are valued ingredients of food or special dishes in the tradition of many people around the world. Documented data indicate that intake of wild-growing mushrooms could exceed 20 kg of fresh product per capita annually in Yunnan province, China, and also in a rural region of the UK (Barret et al. 1999; Zhang et al. 2010). Hence, wild-growing mushrooms without doubt are important organic food items in the diet of many people, while our knowledge on their mineral constituent composition and content, and their fate during culinary processing and their accessibility, has many gaps. In a study of wild-growing mushrooms, it is also important to better understand their mineral constituent composition in the context of the best analytical chemistry, the natural geochemistry of the soil substratum where the mycelium grows as well as the physiology of a species (Aloupi et al. 2011; Falandysz et al. 2012a, b; Kojta and Falandysz 2016; Kojta et al. 2015; Kubrová and Borovička 2015; Mleczek et al. 2016; Nearing et al. 2014; Tel et al. 2014). This study aimed to update information on the content of some essential and hazardous metallic elements accumulated in *Cantharellus* mushrooms foraged in Poland and China as determined by inductively coupled plasma mass spectroscopy with a dynamic reaction cell (ICP-DRC-MS). Available data on the baseline content of the geogenic metallic elements and metalloids in soils from the background areas in Yunnan as well as a geogenic and anthropogenic sources in Poland can explain results obtained for chanterelle mushrooms.

## Materials and methods

### Mushrooms

**Fig. 1:**
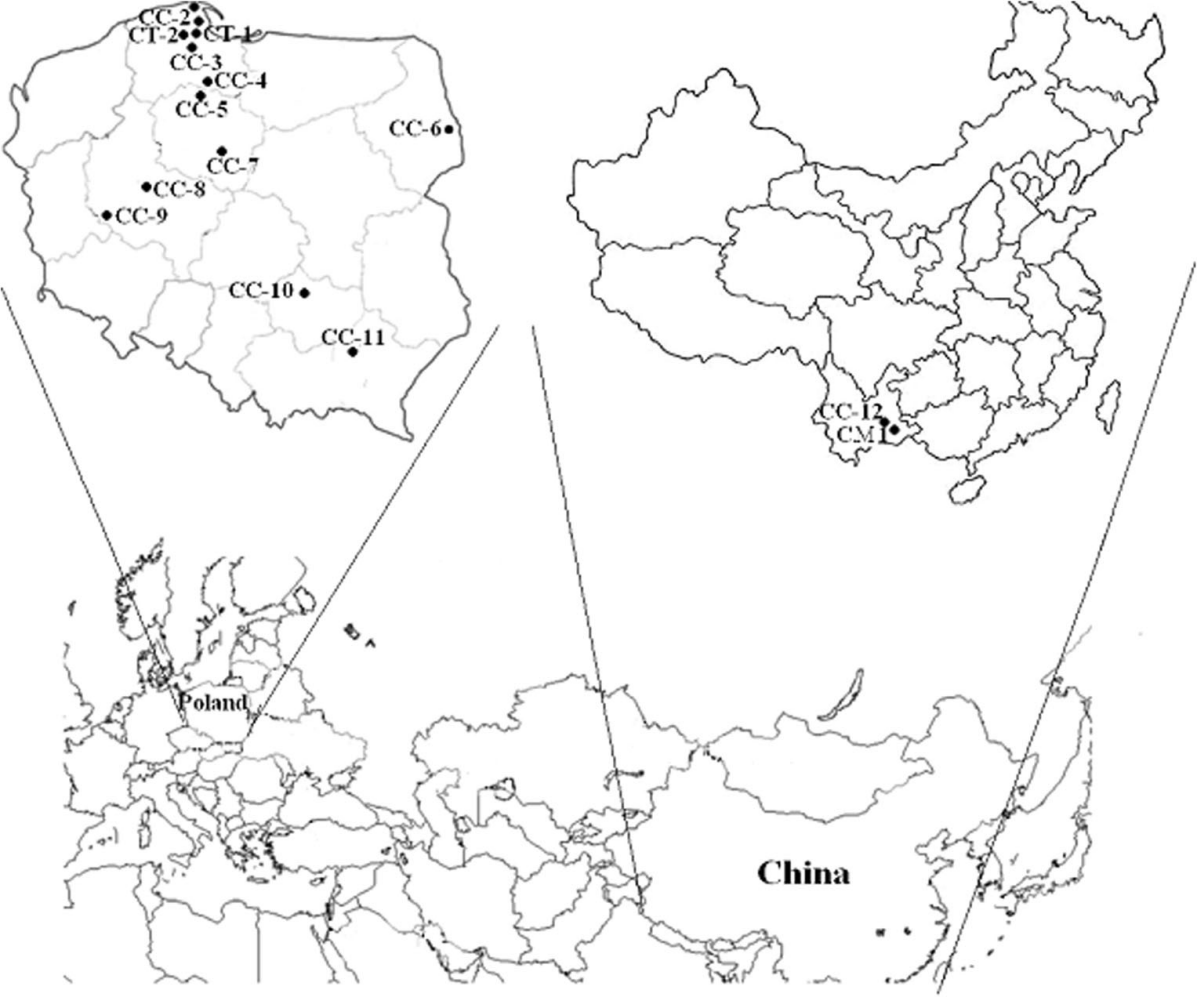
Localization of the sampling places of the *Cantharellus* mushrooms in Poland and China (see Table 2)

Ten composite samples of fruiting bodies of the mushroom *C. cibarius* Fr. were collected from the following sites: Jastrzebia Góra (CC-1), Darżlubska (CC-2), Kościerzyna forests (CC-3), Tuchola Pinewoods–Osiek (CC-4), and Tuchola Pinewoods (CC-5) in the Pomerania region; Ciechocinek (CC-7) in the Kujawy region; Poznań outskirts (CC-8) in Great Poland Voivodship; Bobrowniki (CC-6) in the Podlasie region; Pieszków (CC-10) in the Świętokrzyskie region; and Głogów Małopolski (CC-11) in Little Poland Voivodship of Poland in 2012–2014; one shipment was bought in a shop in Poland (CC-9) and another one was collected from the region of Yuxi county (CC-12) in the province of Yunnan in China in 2013. *C. minor* Peck was collected from Yuxi (CM-1) during 2013 and *C. tubaeformis* (Fr.) Quél from two locations: Mojusz (CT-1) and Kartuzy (CT-2) in Pomerania, Poland, were collected in 2007–2008 (Fig. 1 and Table 2).

Fresh mushrooms were immediately cleaned from any visible plant vegetation and soil debris with a plastic knife and further rinsed with deionized water, and the bottom part of the stem was cut off. Subsequently, the mushroom samples were placed into plastic trays of an electrically heated commercial dryer (dehydrator for mushrooms, fruits, vegetables, and herbs; model MSG-01; MPM Product, Milanówek, Poland, and Ultra FD1000 dehydrator, Ezidri, Australia) and dried at 65 °C to constant mass. Dried fungal materials were pulverized in a porcelain mortar that was cleaned by hand washing using a laboratory brush, deionized water, and detergent and then further rinsed with distilled water and dried in an electrically heated laboratory dryer at 105 °C. Pulverized mushrooms were kept in brand new sealed polyethylene bags under dry conditions.

### Elemental analysis

Before digestion, the samples were dried in 65 °C for 12 h using an electrically heated laboratory oven. Then, subsamples of dried and powdered mushrooms (∼0.5000 g) were digested with 5 mL of 65% HNO_3_ (Suprapure, Merck, Germany) under pressure in a model Ethos One (Milestone Srl, Italy) microwave oven. The rotor body holds up to ten digestion vessels made of high-purity TFM™ polytetrafluoroethylene (PTFE) with capacity of 50-mL per vessel. The heating program was performed in one step: the power of the process was 1500 W, ramp time 20 min, temperature 200 °C, and hold time 30 min. Blank reagent solutions were prepared in the same way. For every set of ten digested mushroom samples, two blank samples were run. The digests were diluted to 10 mL using deionized water (TKA Smart2Pure, Niederelbert, Germany) in volumetric flasks. If this was necessary, a specific digest was further diluted (about ten times) to keep a linearity of measurements or acid concentration below 3%.

## Instrumentation

Elemental analysis of the elements (Ag, As, Ba, Cd, Co, Cs, Cu, Cr, Li, Mn, Ni, Pb, Rb, Sr, V, Tl, U, and Zn) was carried out using the ELAN DRC II ICP-MS Inductively Coupled Plasma Mass Spectrometer (PerkinElmer, SCIEX, Canada) equipped with a Meinhard concentric nebulizer, cyclonic spray chamber, dynamic reaction cell, Pt cones, and quadruple mass analyzer. For dynamic reactive cell (DRC), as reaction gas, ammonia (99.999% purity ammonia, Linde Gas, Kraków, Poland) to eliminate possible spectral interferences in determination of Cr, Mn, V, and Zn) and oxygen (99.999% purity oxygen, Linde Gas, Kraków, Poland) were used for free from spectral interferences determination of As.

The ICP-MS was run at the following conditions: RF power—1100 W; plasma Ar flow rate—15 L min^−1^; nebulizer Ar flow rate—0.87 L min^−1^ and auxiliary Ar flow rate—1.2 L min^−1^; and lens voltage—(7.5–9.0) V. A mixed standard solution with contents of 10 mg L^−1^ was used (Multielement Calibration Standard 3, Atomic Spectroscopy Standard, PerkinElmer Pure). Moreover, the isotopes of ^45^Sc, ^74^Ge, ^103^Rh, and ^159^Tb prepared from individual solutions with contents of 1000 mg L^−1^ were applied as internal standards in order to effectively correct temporal variations in signal intensity (ICP Standard CertiPUR, Merck, Germany).

Generally, calibration curves for elements were prepared in the range of 0.1 to 50 μg L^−1^. Argon (99.999%) was used as a nebulizer, auxiliary, and plasma gas (Messer, Chorzów, Poland). If concentration of an element measured in a digest exceeded the maximum value for the calibration curve, a digest was diluted respectively and re-examined. All absolute values of the element contents determined and presented for the *Chanterelle* mushrooms are given in mg kg^−1^ dry biomass (db).

### Quality control/quality assurance

The methods of trace element measurement were validated and controlled by preparation of standard solutions; calibration of instruments; and daily runs of blank samples, duplicates, and replicates with each analytical cycle. All samples were analyzed in batches with certified reference materials and blanks. Duplicates and blanks were measured with every set of ten mushroom samples. Evaluation of the accuracy of the analytical method was based on analysis of the two certified reference materials (CRM): mushroom powder IC-CS-M-4, Institute of Nuclear Technology and Chemistry in Warsaw, ICHTJ, Poland, and Oriental Basma Tobacco Leaves, INCT-OBTL-5, Institute of Nuclear Technology and Chemistry in Warsaw, ICHTJ, Poland (Table 1). Precision was calculated as the coefficient of variations (CV) of duplicates. As a result of the analysis, the following measurement precision values were in the range from 1 to 8% for all investigated elements. Finally, the limits of detection, calculated as three standard deviations of seven independent replicates of the reagent blank, were respectively (in μg L^−1^) 0.003 for Ag, 0.03 for As, 0.2 for Ba, 0.008 for Cd, 0.001 for Co, 0.2 for Cr, 0.01 for Cs, 0.15 for Cu, 0.02 for Li, 0.4 for Mn, 0.05 for Ni, 0.07 for Pb, 0.03 for Rb, 0.06 for Sr, 0.001 for Tl, 0.004 for U, 0.09 for V; and 4 for Zn.

**Table 1:** Results of the measurements of accuracy of the analytical data using certificate reference materials “fungal powdered fruiting bodies of *Leccinum scabrum*“ IC–CS–M–4 (*n* = *5*) and Oriental basma tobacco leaves, INCT–OBTL–5 (*n* = *5*)

Analyte	Measured value (mg kg^−1^)	Certified value (mg kg^−1^)	Recovery (%)
Ag^b^	0.062 ± 0.003	0.053 ± 0.011	116
As^a^	0.416 ± 0.018	0.375 ± 0.051	111
Ba^b^	63.2 ± 2.2	67.4 ± 3.8	94
Cd^a^	1.43 ± 0.13	1.33 ± 0.09	107
Co^b^	0.949 ± 0.043	0.981 ± 0.067	97
Cr^a^	1.05 ± 0.11	1.09 ± 0.21	96
Cs^b^	0.261 ± 0.01	0.288 ± 0.02	91
Cu^a^	28.18 ± 0.98	26.37 ± 1.74	107
Li^b^	18.8 ± 0.65	19.3^c^	97
Mn^b^	174 ± 5	180 ± 6	97
Ni^b^	8.94 ± 0.49	8.5 ± 0.49	105
Pb^a^	1.032 ± 0.06	0.998 ± 0.072	103
Rb^b^	18.5 ± 0.6	19.1 ± 1	97
Sr^b^	101 ± 1	105 ± 5	96
Tl^b^	0.044 ± 0.001	0.051^c^	86
U^b^	0.100 ± 0.002	0.113^c^	88
V^b^	3.98 ± 0.10	4.12 ± 0.55	97
Zn^a^	127.8 ± 5.2	120.7 ± 5.9	106

^a^IC-CS-M-4

^b^INCT-OBTL-5; ^c^Information values

^c^Information values

### Statistical analyses

The computer software Statistica, version 10.0 (Statsoft Polska, Kraków, Poland), was used for statistical analysis of data and for graphical presentation of the results of two-dimensional multiple scatter plot relationships between the variables.

## Results and discussion

### Cu, Mn, and Zn

Copper, manganese, and zinc are essential elements both for fungi and humans. For *C*. *cibarius* in Poland, the element Cu ranged from 34 up to 53 mg kg^−1^ db in the Świętokrzyskie Mountain region of Piekoszów, while being lower in Yunnan, i.e., at 31 mg kg^−1^ db. *C. minor* from Yunnan showed the lowest content in this study, i.e., 27 mg kg^−1^ db. The Świętokrzyskie Mts. region in the south-central Poland is an area where copper was mined in the past for almost six centuries in some places, and soil background there can be enriched in Cu and also Cr, Mn, Ni, Zn, As, Cd, Pb, and U (Gałuszka et al. 2015). Hence, a maximum content of Cu determined in *C. cibarius* from the forests in the region of the Świetokrzyskie Mts. can be explained largely by anomalous geochemistry of soil there. Copper as many other metallic elements is also transported from the anthropogenic sources of emission to more or less remote areas with moving air masses, but they are largely deposited locally (Nygård et al. 2012; Steinnes and Friedland 2006). Airborne heavy metals and metalloids when deposited can be retained in soil by adsorption and/or precipitation reactions and not readily available for mushrooms. Airborne deposition of some heavy metals (Zn, Pb, Ni, Cd), as it was measured in the organic (O) and mineral (B) layers of the forest soils, was in the past low in the region of the Świętokrzyskie Mts. (Andersen et al. 1994). Also, airborne heavy metal deposition (Pb and As as the tracers) in the region of northwestern Poland was low in the most recent study (Steinnes and Twardowska 2016).

Two sets of *C*. *tubaeformis* from Poland were similarly high in Cu with 35 to 39 mg kg^−1^ db (Table 2). The absolute values of Cu in *Cantharellus* spp. in this study differed between the sites, while contents were in a narrow range in spite of the geographically scattered origin of mushrooms. The data obtained for Cu for three *Cantharellus* species agree well with most results available for *C*. *cibarius* from the different regions of Europe, i.e., they were at 8.2 ± 1.9 (this result was considered as outlier); 26; 30 ± 3; 32 ± 0; 35; 35; 37, 38, 39 ± 2; 35 ± 8 to 49 ± 4; 42; 46 ± 27; 50; 52 ± 8 to 58 mg kg^−1^ db, and it was 90 ± 5 mg kg^−1^ db in Turkey, 73 mg kg^−1^ db in Mexico, and 24 ± 0 mg kg^−1^ db (only three samples) in USA—as was reviewed recently (Drewnowska and Falandysz 2015; Falandysz and Drewnowska 2015).

**Table 2:** Trace metallic elements and arsenic content (mg kg^−1^ db) in fruiting bodies of *Cantharellus* mushrooms

Species, place and year of collection, and number of individuals (*n*) for composite sample and sample ID^*^	Li	V	Cr	Mn	Co	Ni	Cu	Zn	As	Rb	Sr	Ag	Cd	Ba	Pb	Cs	Tl	U
*Cantharellus cibarius* Fr.																		
Poland, Jastrzębia Góra, 2012 (*n* = *57*)^**^ (CC-1)^*^	0.12	0.035	0.15	19	0.24	0.89	37	88	0.066	300	12	0.075	0.26	0.50	0.17	WD	0.074	0.0015
Poland, Darżlubska Wilderness, 2012 (*n* = *52*) (CC-2)	0.051	0.12	0.14	45	0.74	1.8	41	95	0.11	530	1.6	0.11	0.53	1.2	0.26	WD	0.16	0.0059
Poland, Kościerzyna forests, 2014 (*n* = *21*) (CC-3)	0.10	0.25	0.29	45	0.82	1.3	46	100	0.13	290	1.9	0.49	0.50	3.2	0.66	WD	0.033	0.017
Poland, Tuchola Pinewoods, 2013 (*n* = *27*) (CC-5)	0.058	0.15	0.14	51	0.99	2.0	34	88	0.16	540	1.0	0.082	0.44	1.3	0.39	5.6	0.15	0.0065
Poland, Tuchola Pinewoods, Osiek, 2013 (*n* = *90*) (CC-4)	0.088	0.099	0.12	43	0.70	1.8	37	92	0.14	490	0.73	0.15	0.49	1.4	0.38	2.2	2.2	0.13
Poland, Kujawy land, Ciechocinek, 2014 (*n* = *19*) (CC-7)	0.049	0.10	0.15	28	0.33	0.88	44	96	0.11	200	1.5	0.13	0.23	1.8	0.29	WD	0.069	0.0029
Poland, Poznań outskirts, 2014 (*n* = *18*) (CC-8)	0.092	0.15	0.18	40	0.43	1.6	41	99	0.089	490	1.3	0.17	0.31	2.4	0.50	WD	0.061	0.011
Poland, Podlasie, Bobrowniki, 2014 (*n* = *47*) (CC-6)	0.027	0.080	0.074	45	0.39	1.3	34	88	0.10	550	1.3	0.14	0.26	1.6	0.39	WD	0.12	0.0027
Poland, Głogów Małopolski, 2014 (*n* = *27*) (CC-11)	0.065	0.16	0.16	41	1.1	2.2	40	87	0.21	400	1.8	0.23	0.82	2.1	0.49	WD	0.12	0.0042
Poland, Świętokrzyskie , 2014 (*n* = *15*) (CC-10)	0.036	0.088	0.095	22	0.38	0.93	53	91	0.21	370	0.97	0.54	1.6	2.1	0.45	WD	0.35	0.0031
*Median value for 10 sites in Poland*	*0.061*	*0.11*	*0.14*	*42*	*0.56*	*1.4*	*40*	*91*	*0.12*	*490*	*1.5*	*0.15*	*0.46*	*1.7*	*0.39*	*3.9*	*0.12*	*0.0050*
*Mean value for 10 sites in Poland*	*0.069*	*0.12*	*0.15*	*38*	*0.61*	*1.5*	*41*	*92*	*0.13*	*420*	*2.4*	*0.21*	*0.54*	*1.8*	*0.40*	*3.9*	*0.33*	*0.018*
*± S.D. value for 10 sites in Poland*	*0.030*	*0.06*	*0.06*	*11*	*0.30*	*0.5*	*6*	*5*	*0.05*	*120*	*3.4*	*0.17*	*0.41*	*0.7*	*0.14*	*-*	*0.66*	*0.039*
Poland, from shop, 2013 (*n* = *309*) (CC-9)	0.055	0.11	0.27	36	0.45	1.6	42	98	0.12	410	1.4	0.24	0.25	1.8	0.33	WD	0.060	0.0038
China, Yuxi, 2013 (*n* = *33*) (CC-12)	0.15	0.63	0.61	19	0.37	1.1	31	76	0.75	61	5.3	0.25	0.58	9.1	1.1	0.71	0.037	0.034
^a^ *Cantharellus minor* Peck																		
China, Yuxi, 2013 (*n* = *153*) (CM-1)	0.11	0.88	0.47	22	0.60	0.58	27	98	1.2	64	4.2	1.0	2.5	5.4	4.4	0.21	0.074	0.031
*Cantharellus tubaeformis* (Fr.) Quél																		
Poland, Pomerania, Mojusz, 2007 (*n* = *54*) (CT-1)	0.041	0.18	0.12	46	0.076	0.45	39	72	0.19	320	1.1	0.55	0.75	2.2	0.72	2.2	0.094	0.0020
Poland, Pomerania, Kartuzy, 2008 (*n* = 7*1*) (CT-2)	0.030	0.10	0.081	39	0.046	0.41	35	56	0.29	350	0.86	0.096	0.44	1.4	0.64	2.5	0.066	0.0030

*WD* without data; ID^*^ (see Figure 1); ^**^(number of fruiting bodies in a pool)

^a^Synonymy: *Cantharellus minor f. intensissimus* R.H. Petersen; *Cantharellus minor* Peck, *F. minor*; *Merulius minor* (Peck) Kuntze

Mushrooms vary in Cu content sequestered in fruiting bodies because of species-specific requirements, while geochemistry of the soil substratum or anthropogenic pollution may also be factors, which could cause a deficit or surplus of the element (Falandysz et al. 2001; Jorhem and Sundström 1995; Mleczek et al. 2015). A previous study showed that soil substratum geochemistry could have an impact on the quantity of Cu sequestered by *C*. *cibarius* in fruiting bodies; e.g., individuals from the Baltic Sea coastal area with sandy soil bedrock at the Hel Peninsula in Poland contained this metal at 30 ± 3 mg kg^−1^ db, while mushrooms from a region richer in Cu, the montane soils near the town of Zakopane in the Tatra Mts., contained Cu at 52 ± 8 mg kg^−1^ db (Falandysz et al. 2012a). *C. cibarius* was able to absorb Cu more efficiently from the soil substratum poorer in this element (Falandysz and Drewnowska 2015). For soil substrata from background areas unpolluted with Cu, as this was the case in this study, the fruit bodies of *C*. *cibarius* showed to some degree physiological regulation in a sequestration of the element into the fruiting bodies.

The content of Mn in the *Cantharellus* mushrooms was at 19 ± 1 to 51 ± 2 mg kg^−1^ db in *C*. *cibarius* from Poland and at 19 mg kg^−1^ db in fruit bodies from Yunnan, at 22 mg kg^−1^ db in *C. minor*, and at 39 to 46 mg kg^−1^ db in *C*. *tubaeformis*. *Cantharellus cibarius* from the Baltic Sea coastal place near the location of Jastrzębia Góra contained Mn at 19 and 21 ± 5 mg kg^−1^ db—similar to fruit bodies from a nearby costal location at the Hel Peninsula that showed 21 ± 5 mg kg^−1^ db (Falandysz et al. 2012a). Both results suggest on a poor status of Mn in soils at the site of Jastrzębia Góra (for the Hel site Mn was at 2.3 ± 0.2 mg kg^−1^ dry matter) (Falandysz et al. 2012a). Manganese in *C*. *cibarius* and *C. minor* from Yunnan was within the lower range of the contents reported internationally for *C*. *cibarius* (Falandysz and Drewnowska 2015). Manganese, like some other essential mineral constituents in mushrooms, undergoes physiological regulation, and the lower values reported for Yunnan and two locations in Poland (the coastal and an upland—Świętokrzyskie mountains) can imply an insufficient supply of the element from the soil substrate.

Zinc in *C*. *cibarius* foraged in Poland was at very similar contents for the sites, i.e., at 88 to 100 mg kg^−1^ db and at 76 mg kg^−1^ db for Yunnan. In *C. minor*, Zn was at 98 mg kg^−1^ db and in *C*. *tubaeformis* at 56 to 72 mg kg^−1^ db. Those values on Zn contents are in the central to upper range of the literature values reported for *C*. *cibarius* (Falandysz and Drewnowska 2015).

### Co, Cr, and Li

Cobalt, chromium, and lithium are considered as minor trace elements in mushrooms (Řanda and Kučera 2004; Vetter 2005). The examined *C. tubaeformis* contained cobalt at 0.046 to 0.076 mg kg^−1^ db. Cobalt in *C. minor* and *C. cibarius* was at an order of magnitude greater than in *C. tubaeformis*, i.e., at 0.24 to 1.1 mg kg^−1^ db (Table 2); those values agree well with data reported for *C. cibarius* foraged in Poland and examined by ICP-optical emission spectroscopy (OES) (Falandysz and Drewnowska 2015).

*C. cibarius* and *C. minor* from Yunnan had chromium at 0.47 to 0.61 mg kg^−1^ db and were richer (*p* < 0.05) in this element than were *C. cibarius* and *C. tubaeformis* from Poland, which contained it at 0.074 to 0.29 mg kg^−1^ db (Table 2). This could be explained by the specific geochemistry of soils in Yunnan, where bedrock in the Circum-Pacific mercuriferous belt is polymetallic and enriched in Cr (Fan 1991).

Chromium, because of the spectral interferences from other ions, which are formed in a stream of atomized ions in the air-acetylene flame or an argon plasma, is difficult or impossible to determine credibly as trace element in biological matrices like mushrooms by flame atomic absorption spectroscopy (F-AAS), ICP-OES, or ICP-MS without a dynamic reaction cell. This could have caused doubtful results published on Cr in mushrooms in the past. The use of a dynamic reaction cell overcomes this problem. The results on chromium in *C. cibarius* and *C. tubaeformis* foraged in Poland, when determined by ICP-MS using a dynamic reaction cell, are within range of data for this element in *C*. *cibarius* at 0.14 ± 0.02 to 0.16 ± 0.03 mg kg^−1^ db when determined by ICP-OES (Falandysz and Drewnowska 2015).

There is a scarcity of data on lithium in mushrooms. In a study by Vetter (2005), the content of Li in mushrooms in Hungary was well below 1.0 mg kg^−1^ db. Also, *Sarcodon imbricatus* (L.) P. Karst. sampled in Poland contained only a small amount of Li, i.e., at 0.016 ± 0.004 in caps and at 0.054 ± 0.035 mg kg^−1^ db in stems (Mędyk et al. 2017).

There is no previous analytical information on Li in *C. cibarius*, *C. tubaeformis*, and *C. minor*. The range for Li in *C. cibarius* from Poland was from 0.027 to 0.12 mg kg^−1^ db, and those data confirm the suggestion that mushrooms foraged in Europe are poor in this element. The result for a sole composite sample of the Yunnan’s *C. cibarius* is at 0.15 mg kg^−1^ db of Li, which is roughly threefold greater than the median value of 0.059 mg kg^−1^ db for individuals from Poland.

An examination by Yin et al. of some mushrooms foraged in the northern and northwestern regions of Yunnan showed lithium at ∼20- to ∼100-fold greater contents than in the mushrooms in this study (Yin et al. 2012). A sample of *C. minor* Peck, from the Dayao county in Yunnan, showed lithium at 2.6 mg kg^−1^ db. Some other mushrooms foraged showed lithium at >1 mg kg^−1^ db: for example, in *Thelephora vialis* Schwein., it was 12 mg kg^−1^ db; in *Tricholoma matsutake* (S. Ito & S. Imai) Singer, 10 mg kg^−1^ db; in *Amanita exitialis* Zhu L. Yang & T.H. Li, 4.9 mg kg^−1^ db; and in *Russula lepida* Fr., current name *R. rosea* Pers., 4 mg kg^−1^ db (Yin et al. 2012).

### Cs and Rb

Cesium was determined only in a few samples in this study and *C. cibarius* from Poland and *C. tubaeformis* showed this element at 2.2 and 5.6 mg kg^−1^ db, and an order of magnitude smaller values were found in Yunnan’s *Cantharellus*, which showed from 0.21 to 0.71 mg kg^−1^ db (Table 2). Because of the nuclear weapon use and tests and the accidents in the Chernobyl nuclear power plant, the *Cantharellus* spp. and other mushrooms in Poland, while far less in Yunnan, can be contaminated with radioactive ^137^Cs (Falandysz et al. 2016). Hence, ^137^Cs in the *Cantharellus* spp. from Poland certainly contributed to total Cs as was measured by ICP-MS.

Also, rubidium was found at an order of magnitude greater content in *C. cibarius* and *C. tubaeformis* collected in Poland (200 to 550 and 320 to 540 mg kg^−1^ db), when related to *C. cibarius* and *C. minor* from Yunnan, which contained 61 and 64 mg kg^−1^ db. The smaller content of Cs and Rb in *Cantharellus* spp. in Yunnan could be related to the geochemical background condition of soils, which are highly weathered in Yunnan (He et al. 2004).

### Ba and Sr

The *Cantharellus* spp. from Yunnan, when compared to *Cantharellus* spp. from Poland, could be considered as enriched in barium and strontium (Table 2). Barium in Yunnan was at 5.4 to 9.1 mg kg^−1^ db and in Poland at <2 mg kg^−1^ db (median value) and strontium at 4.2 to 5.3 mg kg^−1^ db and at <2 mg kg^−1^ db. An exception was *C. cibarius* from the site in Jastrzębia Góra in the Baltic Sea coastal area in Poland, which contained Sr at 12 mg kg^−1^ db (Table 2). Both Ba and Sr may be enriched in the polymetallic soils of Yunnan due to a geochemical anomaly (Fan 1991). This could have an impact on occurrence of Ba and Sr in mushrooms in Yunnan.

Data are lacking on efficiency of bioconcentration by mushrooms of Ba and Sr from soils poor in Ca. Certain mushrooms, e.g., stinkhorns such as *Phallus impudicus* L. and *Clathrus ruber P. micheli* ex Pers., have much calcium, which is used to stabilize the gelatinous layer protecting the embryonal carpophore, and they also concentrate Ba and Sr (T. Stijve, personal information). Also, some slime molds, e.g., *Fuligo septica* (L.) Wiggers, can well accumulate Ca, Ba, and Sr (Setala and Nuorteva 1989; Stijve et al. 1999).

Barium at 14 mg kg^−1^ db and strontium at 2.8 mg kg^−1^ db were at elevated levels also in *C. minor* from Yunnan (Yin et al. 2012). *Boletus* species foraged in Yunnan also showed an elevated content of ^90^Sr (and a very low amount of ^137^Cs) from radioactive fallout, when related to individuals foraged in Poland (Falandysz et al. 2016; Saniewski et al. 2016).

### Ni, V, and U

Nickel was at similar contents in the *Cantharellus* mushrooms in Poland (range from 0.88 to 2.2 mg kg^−1^ db) and Yunnan (0.58 to 1.1 mg kg^−1^ db) (Table 2). *C. cibarius* foraged in several places in Poland when examined by ICP-OES showed Ni from 0.77 ± 0.22 to 2.0 ± 0.6 mg kg^−1^ db (Falandysz and Drewnowska 2015). Data on Ni in *C. cibarius*, *C. minor*, and *C. tubaeformis* obtained by ICP-MS (Table 2) confirmed that the content of this element in *Cantharellus* mushrooms rarely exceeds 2.0 mg kg^−1^ db, as was indicated by the ICP-OES analyses (Falandysz and Drewnowska 2015).

Vanadium was found at a greater content in *C. cibarius* and *C. minor* mushrooms in Yunnan (ranging from 0.63 to 0.88 mg kg^−1^ db) than in Poland (ranging from 0.035 to 0.25 mg kg^−1^ db) (Table 2). Vanadium is one of the metallic elements that are enriched in red and yellow soils of Yunnan (Fan 1991). *C. cibarius* sampled from the paleozolic graywacke bedrock in Western Bohemia contained vanadium at 0.21 ± 0.04 mg kg^−1^ db (Řanda and Kučera 2004), which is similar to the maximum value of 0.25 mg kg^−1^ db determined in *C. cibarius* from a site in Poland in this study. The median value for mushrooms from Poland was at 0.11 mg kg^−1^ db (Table 2).

Uranium was at 0.031 to 0.034 mg kg^−1^ db in the *Cantharellus* mushrooms in Yunnan and at 0.0015 to 0.13 mg kg^−1^ db in Poland (Table 2). Those values for *Cantharellus* mushrooms are close to the median value of 0.063 mg kg^−1^ db and are within the range for uranium in ectomycorrhizal macro fungi growing in an unpolluted area (Kubrová and Borovička 2015).

### Ag, As, Cd, Pb, and Tl

Silver in *C. cibarius* was at 0.075 to 0.54 mg kg^−1^ db, in *C. minor* at 1.0 mg kg^−1^ db, and in *C. tubaeformis* at 0.096 to 0.55 mg kg^−1^ db (Table 2). Those values agree with what was noted in *C. cibarius* from numerous places in Poland and in Czech Republic, which contained Ag at 0.60 ± 0.21 mg kg^−1^ dm, from 0.050 ± 0.017 to 0.19 ± 0.04 mg kg^−1^ dm, and at 0.17 ± 0.04 mg kg^−1^ dm, when examined respectively by F-AAS, ICP-OES, and neutron activation analysis (Falandysz and Drewnowska 2015; Falandysz et al. 1994; Řanda and Kučera 2004).

Arsenic in *C. cibarius* from Poland was at 0.066 to 0.21 mg kg^−1^ db and in *C. tubaeformis* at 0.19 to 0.29 mg kg^−1^ db, while mushrooms from Yunnan showed greater contents: 0.75 mg kg^−1^ db in *C. cibarius* and 1.2 mg kg^−1^ db in *C. minor*. Deposition of airborne As in forests in the northwestern part of Poland is low (Steinnes and Twardowska 2016). Mushrooms from the forested areas with geochemical anomaly in Poland can be richer in geogenic As when related to those foraged from a typical background areas. An example was mushroom *Amanita fulva* from the Lower Silesia forest in region of a geochemical anomaly in SW Poland having As at 0.78 ± 0.05 mg kg^−1^ db in caps and 1.3 ± 0.1 mg kg^−1^ db in stipes (Falandysz et al. 2017). Arsenic content was also low (0.36 ± 0.02 mg kg^−1^ db) in *C. cibarius* from Western Bohemia (Řanda and Kučera 2004).

The laterite red earths and red earths and yellow earths of Yunnan are enriched in the metalloids As and Sb (Fan 1991). This could explain a somewhat elevated content of As in *Cantharellus* mushrooms in Yunnan in this study while levels were relatively low (Table 2).

Cadmium was relatively well studied in *C. cibarius* in Europe, while there is lack of comprehensive information on the occurrence of this heavy metal in material from Yunnan in China (Falandysz and Drewnowska 2015). Cadmium in portion is leaching out when *C. cibarius* is blanched (Unpublished; JF). This metallic element occurred at 1.6 mg kg^−1^ db in *C. cibarius* from the Pieszków site in the Świętokrzyskie Mountains region, which could be considered as elevated compared to the range from 0.23 to 0.82 mg kg^−1^ db for other places in Poland in this study (Table 2). The median value of Cd in *C. cibarius* for a range of sites in Poland was determined in an earlier study at 0.15 to 0.36 mg kg^−1^ db (Falandysz and Drewnowska 2015). The Świętokrzyskie Mountains area is enriched in some metal ores and also with a long history of some metallurgic industrial activities in certain sites (Gałuszka et al. 2015). Hence, as it was in the case of copper, an overriding source of Cd in *C. cibarius* from the Świętokrzyskie Mountains region can be geogenic than anthropogenic Cd.

Cadmium in *C. cibarius* in Yunnan was at 0.58 mg kg^−1^ db and somewhat higher in *C. minor*, i.e., at 2.5 mg kg^−1^ db. In a sample of *C. minor* from the northern region of Yunnan, Yin et al. (2012) found this element at 1.6 mg kg^−1^ db. Cd in *C. tubaeformis* was similar to its content in *C. cibarius* and from 0.44 to 0.75 mg kg^−1^ db. Cadmium, in a recent study from NW Spain, was found in the gills of *C. cibarius* at 0.43 ± 0.19 mg kg^−1^ db (0.17–0.71; *n* = *13*) and in the main part of fruiting bodies at 0.25 ± 0.12 mg kg^−1^ db (0.31–1.2) (Melgar et al. 2016).

Lead in *C. cibarius* and *C. tubaeformis* from Poland was respectively at range 0.17–0.66 and 0.64–0.72 mg kg^−1^ db and similar to *C. cibarius* in Yunnan, i.e., at 1.1 mg kg^−1^ db. Pollution with the airborne lead of the forested areas in Poland outside of large local emitters was considered low in the past (Andersen et al. 1994) and recently (Steinnes and Twardowska 2016). In light of the data for *C. cibarius*, *C. minor* from Yunnan with lead at 4.4 mg kg^−1^ db could be considered as contaminated. The species *C. minor* from the northern region of Yunnan showed lead at 2.6 mg kg^−1^ db (Yin et al. 2012).

There is no earlier information on the occurrence of thallium in *Cantharellus* mushrooms from Poland or Yunnan. In this study, *C. cibarius* from the Osiek site in the Tuchola Pinewoods contained thallium at 2.2 mg kg^−1^ db, which is an elevated level when compared to *C. cibarius* sampled elsewhere in Poland (range 0.033 to 0.35 mg kg^−1^ db), and also to *C. tubaeformis* (range from 0.066 to 0.094 mg kg^−1^ db) (Table 2). A reason of an elevated content of Tl in *C. cibarius* from the Tuchola Pinewoods is unknown since no data on its content and origin in soil there can be available. Data on thallium in forest soil of Poland are lacking, while concentrations of Tl extracted by EDTA in soils of other types were low, i.e., in range 0.1–0.4 mg kg^−1^, and elevated in soils from a site close to the zinc ore mine waste dumping site (Łukaszewicz and Zembrzuski 1992).

Several species of mushrooms sampled in Poland when examined by high-resolution ICP-MS showed thallium at <0.1 mg kg^−1^ db (Falandysz et al. 2001).

### Multivariate analysis

**Fig. 2:**
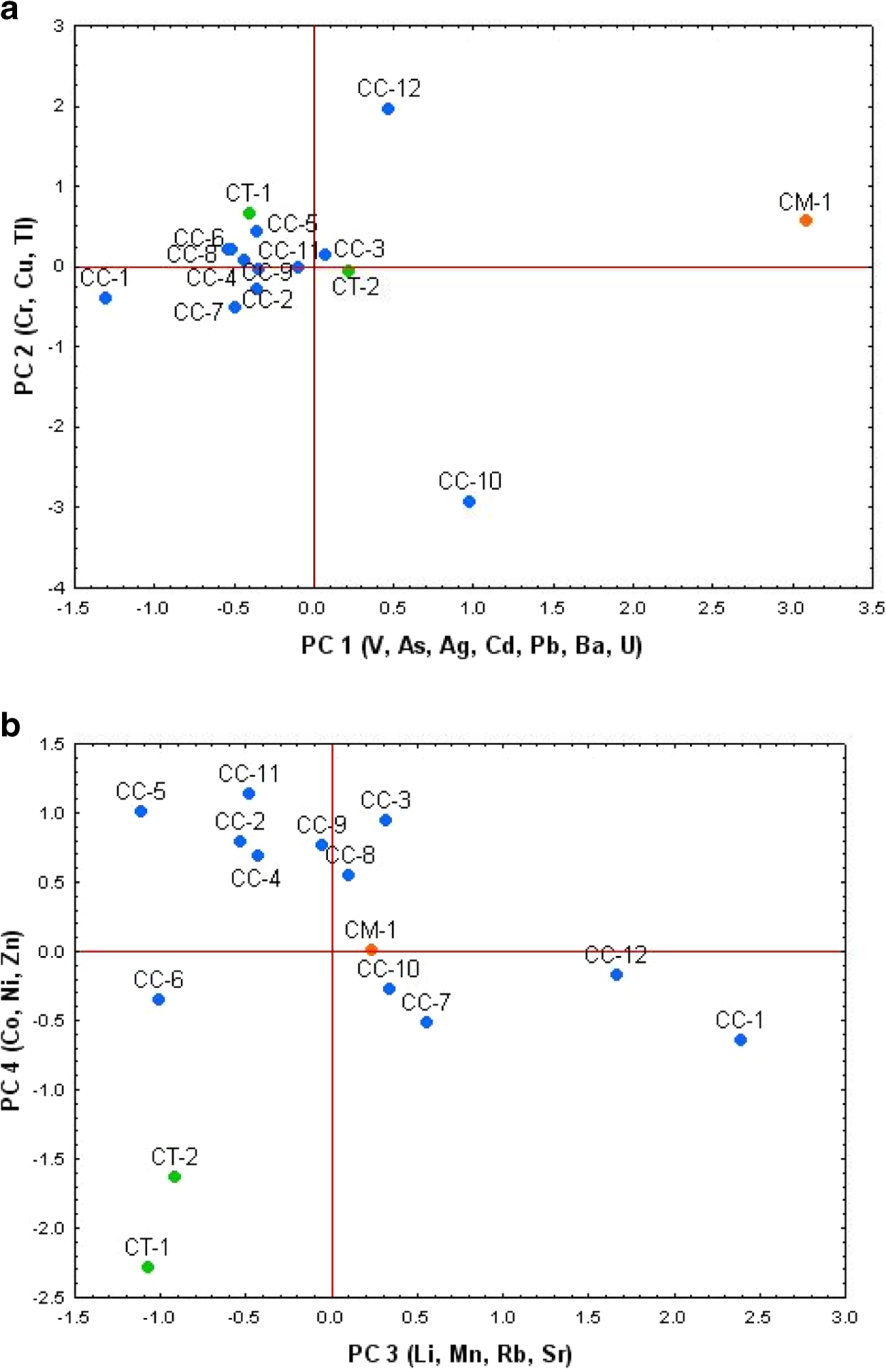
Principal component analysis of the trace metallic elements and arsenic in *Cantharellus* mushrooms and places of collection (**a** PC1/PC2; **b** PC3/PC4). Associations among the places are shown on PC 1 and PC2 (**a**) and PC3 and PC4 with Varimax unrotated matrixes

A multivariate approach and applying the principal component analysis (PCA) (Chudzińska and Barałkiewicz 2010; Wyrzykowska et al. 2001) has been used to examine the correlation matrix from a possible 17 × 15 data matrix (17 elements sequestered in fruiting bodies by *C. cibarius* (CC), *C. minor* (CM), and *C. tubaeformis* (CT) from 15 places) but without cesium that was determined only in six samples), and results are presented in Table 3 and Fig. 2. All data were standardized to bring values to compatible units from a distribution with a mean of 0 and a standard deviation of 1. In choosing the number of components, the Kaiser criterion (factors with eigenvalues greater than 1) and scree test were used. Varimax normalized rotation was used in order to maximize the variances of normalized factor loadings across variables for each factor. Loadings values of >0.75 are considered strong, between 0.75 and 0.5 moderate, and 0.5 and 0.3 weak based on their absolute values. In this study, only component loadings ≥0.55 were taken for interpretation. The PCA examination of data (absolute concentration values and sampling places) revealed that 85% of information regarding the mineral compositional variability in mushroom samples for all examined sites could be described by four components.

**Table 3:** Factor loadings (varimax normalized rotation)

Eigenvalues	5.7	3.1	3.1	2.5
Total variance (%)	34	18	18	15
Cumulative (%)	34	52	70	85
Variables	PC1	PC2	PC3	PC4
*Li*	0.19	0.47	*0.75* ^#^	*0.26*
*V*	*0.83*	0.49	0.25	0.06
*Cr*	0.53	*0.55*	0.49	0.21
Mn	−0.37	0.13	*−0.81*	0.28
*Co*	0.08	0.05	−0.21	*0.90*
*Ni*	−0.35	0.04	−0.22	*0.87*
Cu	−0.25	*−0.79*	0.04	0.14
*Zn*	0.10	−0.28	0.21	*0.77*
*As*	*0.87*	0.40	0.20	−0.10
Rb	*−0.57*	−0.28	*−0.59*	0.33
*Sr*	0.07	0.14	*0.83*	−0.13
*Ag*	*0.92*	−0.13	0.05	−0.06
*Cd*	*0.94*	−0.22	0.03	−0.01
*Ba*	*0.57*	0.53	0.39	0.02
*Pb*	*0.90*	0.26	0.077	−0.04
Tl	0.13	*−0.80*	−0.21	0.11
U	*0.66*	0.55	0.38	0.16

^#^strong interreletion (in Italics)

The component loadings showed that the PC1 explained 34% of the total variance and loaded on the positively and negatively (for Rb) correlated variables, describing significant association between V, As, Ag, Cd, Pb, Ba, and U in chanterelles independently of the species and place. The PC2 was loaded primarily by positively correlated Cr and negatively correlated Cu and Tl and accounted for 18% of the total variance. The PC3 was correlated (negatively) with Mn and Rb and (positively) with Li and Sr and explained 18% of the total variance, while PC4, which positively correlated with Co, Ni, and Zn, accounted for 15% of the total variance.

A projection of datasets on the factor plane allowed the visualization of the relationship between the trace element contents of the samples and the sampling places (Fig. 2). Projection of data on the PC1 and PC2 plane separated a sample of *C. minor* (CM-1) from China, which indicated the highest contents of V, As, Ag, Cd, Pb, Ba, and U while lowest of Rb, while *C. cibarius* (CC-1) from the coastal region of the Jastrzębia Góra site in the Baltic Sea costal area in Poland was lowest in those elements except of Rb (Fig. 2a). Next, *C. cibarius* (CC-12) from China separated by PC2 had the highest content of Cr with the lowest content of Cu and Tl, while *C. cibarius* (CC-10) from the Piekoszów site in the Świętokrzyskie Mountains with a highest content of Cu and elevated Tl was relatively poor in Cr (Fig. 2a). Projection of data on the PC3 and PC4 plane (Fig. 2b) separated *C. cibarius* (CC-12) from China and Jastrzębia Góra (CC-1), because of the higher content of Li and Sr and lower of Mn and Rb. Also, *C. tubaeformis* (CT-1 and CT-2) from both sites separates from other collections (negative correlation), because of lower contents of correlated Co, Ni, and Zn in its fruiting bodies, while a positive tendency could be observed between those elements in *C. cibarius* from three sites in Poland (CC-5 for Tuchola Pinewoods, CC-11 for Głogów Małopolski, and CC-3 for Kościerskie forests).

Since total concentrations of the elements were determined, the associations found can be largely explained by considering that the mineral content of mushrooms depends on the (i) geochemical composition of soil bedrock—as was observed for lithophile elements such as As, Ba, Cr, Li, Sr, V, and U which were relatively abundant in chanterelles but are known as abundant in red and yellow earths of Yunnan or were lower (Mn, Rb) in mushrooms from Yunnan, (ii) chalcophilicity of certain elements (Ag, As, Cd, Pb), and (iii) essentiality concentrations—adequate supplementation and sometimes deficiency or excess (Cu, Co, Mn, Ni, Zn).

## Conclusions

This study filled some information gaps on the mineral constituents of *C. cibarius* foraged in Poland and in Yunnan, China. Provided were for first time and discussed data on As, Li, V, Tl, and U in chanterelles from Poland and on Ba, Co, Cr, Ni, Rb, and Sr in chanterelles from China. The results obtained suggest a strong influence of the collection site on the occurrence of As, Ba, Cr, Li, Mn, Pb, Rb, Sr, U, and V in fruit bodies. These elements were found at higher values in *Cantharellus* mushrooms from polymetallic soils of Yunnan in China, but lower values were measured for Cs, Mn, and Rb, when compared to fruit bodies from Poland.
